# Supplementary material for: Chronic sleep deprivation is associated with delayed puberty onset in rats, activation of proinflammatory cytokines and gut dysbiosis
Source: PeerJ. 2025 Jul 9;13:e19668. doi: 10.7717/peerj.19668 (PMC12255245; doi:10.7717/peerj.19668)
Supplement: Supplemental Information 1 — Data are reported as mean ± SEM (female, n = 6 rats/group; male, n = 6 rats/group ), and values with different superscripts are significantly different compared to the control group (p < 0.05). Abbreviations: TP, Total Protein; AST(GOT), aspartate aminotransferase (glutamic-oxaloacetic transaminase); ALT(GPT), alanine transaminase (glutamic pyruvic transaminase); ALKP, alkaline phosphatase, BUN, blood urea nitrogen; CRE, creatinine; TG, triglycerides; CHOL, cholesterol; HDL, high-density lipoprotein; LDL, low-density lipoprotein; PND, post-natal day; CF, control female; SDF, sleep deprivation female; CM, control male; SDM, sleep deprivation male. [file peerj-13-19668-s001.docx]

**Table S1.** Basic biochemical characteristics

| **Biochemical characteristics** | **Group** | | | |
| --- | --- | --- | --- | --- |
|  | **Female** | | **Male** | |
|  | **CF** | **SDF** | **CM** | **SDM** |
| TP (mg/dL) | 5.40±0.04 | 5.13±0.84* | 5.42±0.03 | 4.398±0.07* |
| Albumin (mg/dL) | 3.23±0.04 | 3.03±0.06* | 3.24±0.03 | 2.94±0.04* |
| AST(GOT) (u/L) | 73.50±4.62 | 81.67±6.01 | 101.83±3.22 | 120.33±4.80* |
| ALT(GPT) (u/L) | 213.5±17.98 | 232.67±20.69 | 41.50±0.62 | 40.50±2.00 |
| ALKP (u/L) | 214.38±16.10 | 232±15.57 | 410.83±26.77 | 358.83±28.48 |
| BUN (mg/dL) | 13.63±1.48 | 14.02±1.37 | 13.65±1.01 | 14.70±0.69 |
| CRE (mg/dL) | 0.17±0.01 | 0.16±0.01 | 0.21±0.02 | 0.16±0.01* |
| Glucose (mg/dL) | 140.83±13.33 | 135.17±13.56 | 171.33±8.74 | 95.83±9.32* |
| TG m(g/dL) | 32.50±3.64 | 24.17±1.97 | 41.83±2.36 | 64.50±7.86* |
| CHOL (mg/dL) | 69.67±3.38 | 62.00±2.29 | 49.50±2.32 | 51.33±3.57 |
| HDL (mg/dL) | 23.40±0.74 | 23.20±1.19 | 21.35±0.97 | 21.60±0.92 |
| LDL (mg/dL) | 4.83±0.75 | 3.83±1.11 | 7.00±0.45 | 4.17±0.31* |

Data are reported as mean ± SEM (female, n = 6 rats/group; male, n = 6 rats/group), and values with different superscripts are significantly different compared to the control group (*p* < 0.05). Abbreviations: TP, Total Protein; AST(GOT), aspartate aminotransferase (glutamic-oxaloacetic transaminase); ALT(GPT), alanine transaminase (glutamic pyruvic transaminase); ALKP, alkaline phosphatase, BUN, blood urea nitrogen; CRE, creatinine; TG, triglycerides; CHOL, cholesterol; HDL, high-density lipoprotein; LDL, low-density lipoprotein; PND, post-natal day; CF, control female; SDF, sleep deprivation female; CM, control male; SDM, sleep deprivation male.
