# Supplementary material for: Chronic sleep deprivation is associated with delayed puberty onset in rats, activation of proinflammatory cytokines and gut dysbiosis
Source: PeerJ. 2025 Jul 9;13:e19668. doi: 10.7717/peerj.19668 (PMC12255245; doi:10.7717/peerj.19668)
Supplement: Supplemental Information 2 — Data are reported as mean ± SEM (female, n = 6 rats/group; male, n = 6 rats/group), and values with different superscripts are significantly different compared to the control group (p < 0.05) . Abbreviations: PRAT, perirenal adipose tissue; eWAT, epididymal white adipose tissue; CF, control female; SDF, sleep deprivation female; CM, control male; SDM, sleep deprivation male. [file peerj-13-19668-s002.docx]

**Table S2.** Retrieved organ weight

| **Organ weight (gram)** | | | | |
| --- | --- | --- | --- | --- |
|  | **Female** | | **Male** | |
|  | **CF** | **SDF** | **CM** | **SDM** |
| Brain | 1.74±0.36 | 1.80±0.04 | 1.85±0.02 | 1.81±0.02* |
| Muscle | 2.43±0.72 | 2.07±0.43* | 3.52±0.07 | 2.61±0.11* |
| PRAT | 1.10±0.21 | 0.68±0.13 | 1.17±0.12 | 0.38±0.09* |
| eWAT |  |  | 1.80±0.13 | 1.12±0.08* |
| Intestine | 6.94±0.40 | 6.85±0.64 | 8.73±0.51 | 7.74±0.21 |
| Colon | 1.14±0.05 | 1.19±0.08 | 2.60±0.19 | 3.66±0.28* |
| Liver | 5.45±0.72 | 5.59±0.25 | 9.05±0.25 | 6.77±0.30* |
| Renal | 0.81±0.25 | 0.75±0.21 | 1.14±0.26 | 0.96±0.22* |
| Ovary | 0.05±0.00 | 0.05±0.00 |  |  |
| Uterus | 0.47±0.11 | 0.40±0.05 |  |  |
| Testis |  |  | 1.36±0.32 | 1.31±0.32 |
| Epididymis |  |  | 0.28±0.01 | 0.24±0.00* |
| Seminal vesicle |  |  | 0.728±0.04 | 0.53±0.06* |

Data are reported as mean ± SEM (female, n = 6 rats/group; male, n = 6 rats/group), and values with different superscripts are significantly different compared to the control group (*p* < 0.05). Abbreviations: PRAT, perirenal adipose tissue; eWAT, epididymal white adipose tissue; CF, control female; SDF, sleep deprivation female; CM, control male; SDM, sleep deprivation male.
