# Supplementary material for: Chronic sleep deprivation is associated with delayed puberty onset in rats, activation of proinflammatory cytokines and gut dysbiosis
Source: PeerJ. 2025 Jul 9;13:e19668. doi: 10.7717/peerj.19668 (PMC12255245; doi:10.7717/peerj.19668)
Supplement: Supplemental Information 3 — a: p-value is determined by Spearman’s correlation analysis ; * p < 0.05. Abbreviations: CF, control female; SDF, sleep deprivation female. [file peerj-13-19668-s003.docx]

**Table S3.** The relationship between abundant bacterial taxa in SDF and CF groups and vaginal opening day

| **Group** | **Taxonomy** | **Spearman’s *rho*** | ***p*-value^a^** |
| --- | --- | --- | --- |
| SDF | g_Muribaculaceae_Uncultured_bacterium | 0.419 | 0.176 |
|  | g_Muribaculaceae_Uncultured_organism | 0.469 | 0.124 |
|  | g_Prevotellaceae_UCG-001 | 0.453 | 0.139 |
|  | g_Ruminococcaceae_UCG-005 | 0.633 | 0.027* |
| CF | g_Ruminococcaceae_NK4A214_group | 0.067 | 0.837 |
|  | g_Roseburia | -0.696 | 0.012* |
|  | g_Lachnospiraceae_GCA-900066575 | -0.384 | 0.218 |
|  | g_Ruminiclostridium_9 | -0.233 | 0.465 |
|  | g_Clostridiales_vadinBB60_group_Uncultured | -0.165 | 0.608 |
|  | g_Lachnospiraceae_Uncultured | -0.551 | 0.063 |
|  | g_Ruminococcus_1 | -0.358 | 0.253 |

a: *p-*value is determined by Spearman's correlation analysis; **p*<0.05. Abbreviations: CF, control female; SDF, sleep deprivation female.
