# Supplementary material for: Chronic sleep deprivation is associated with delayed puberty onset in rats, activation of proinflammatory cytokines and gut dysbiosis
Source: PeerJ. 2025 Jul 9;13:e19668. doi: 10.7717/peerj.19668 (PMC12255245; doi:10.7717/peerj.19668)
Supplement: Supplemental Information 4 — a: p value is determined with Spearman’s correlation analysis ; * p < 0.05; ** p < 0.01. Abbreviations: CM, control male; SDM, sleep deprivation male. [file peerj-13-19668-s004.docx]

**Table S4** The relationship between abundant bacterial taxa in SDM and CM groups and preputial separation day

| **Group** | **Taxonomy** | **Spearman’s rho** | ***p*-value^a^** |
| --- | --- | --- | --- |
| SDM | g_Prevotellaceae_NK3B31_group | 0.587 | 0.045* |
|  | g_Ruminococcaceae_UCG-010 | 0.518 | 0.085 |
|  | g_Eubacterium_coprostanoligenes_group | 0.465 | 0.128 |
|  | g_Shuttleworthia | 0.138 | 0.668 |
| CM | g_Butyricicoccus | -0.562 | 0.057 |
|  | g_Lachnospiraceae_A2 | -0.631 | 0.028* |
|  | g_Ruminococcaceae_UCG-013 | -0.569 | 0.054 |
|  | g_Ruminiclostridium_9 | -0.740 | 0.006** |
|  | g_Clostridium_sensu_stricto_1 | -0.825 | 0.001** |
|  | g_Lactobacillus | -0.546 | 0.066 |
|  | g_Clostridiales_vadinBB60_group_Uncultured | -0.641 | 0.025* |

a: *p* value is determined with Spearman's correlation analysis; **p*<0.05; ***p*<0.01. Abbreviations: CM, control male; SDM, sleep deprivation male.
