# Supplementary figures and images for: Chronic sleep deprivation is associated with delayed puberty onset in rats, activation of proinflammatory cytokines and gut dysbiosis
Source: PeerJ. 2025 Jul 9;13:e19668. doi: 10.7717/peerj.19668 (PMC12255245; doi:10.7717/peerj.19668)

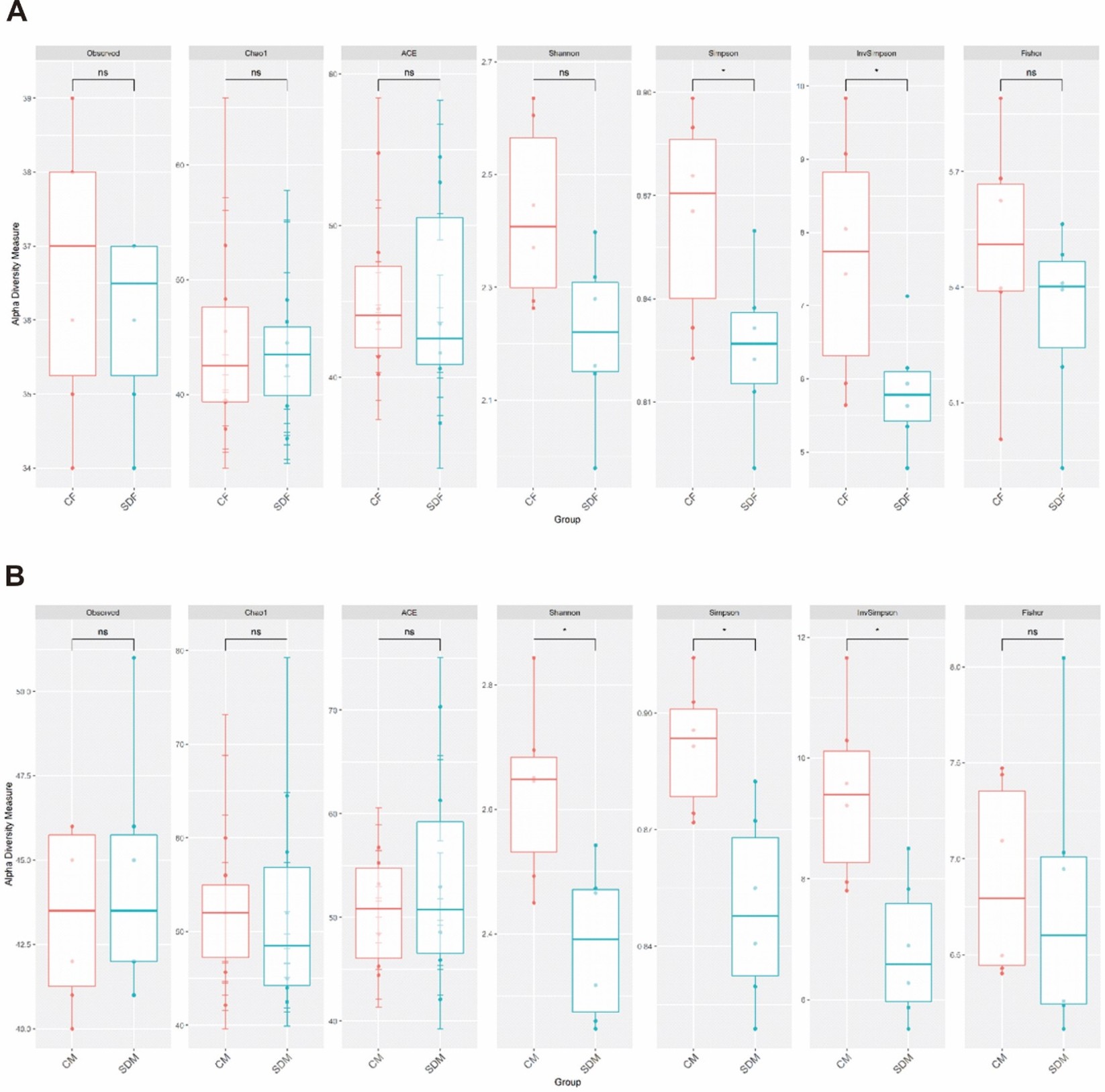

Supplement: Supplemental Information 5 — Abbreviations: CF, control female; SDF, sleep deprivation female; CM, control male; SDM, sleep deprivation male. [file peerj-13-19668-s005.jpg]
